# Empirical validation of the S-Score algorithm in the analysis of gene expression data

**DOI:** 10.1186/1471-2105-7-154

**Published:** 2006-03-17

**Authors:** Richard E Kennedy, Kellie J Archer, Michael F Miles

**Affiliations:** 1 Department of Biostatistics, Virginia Commonwealth University, Richmond, VA 23298, USA; 2 Department of Pharmacology and Toxicology, Virginia Commonwealth University, Richmond, VA 23298, USA; 3 Department of Neurology, Virginia Commonwealth University, Richmond, VA 23298, USA; 4 Center for the Study of Biological Complexity, Virginia Commonwealth University, Richmond, VA 23298, USA

## Abstract

**Background:**

Current methods of analyzing Affymetrix GeneChip^® ^microarray data require the estimation of probe set expression summaries, followed by application of statistical tests to determine which genes are differentially expressed. The S-Score algorithm described by Zhang and colleagues is an alternative method that allows tests of hypotheses directly from probe level data. It is based on an error model in which the detected signal is proportional to the probe pair signal for highly expressed genes, but approaches a background level (rather than 0) for genes with low levels of expression. This model is used to calculate relative change in probe pair intensities that converts probe signals into multiple measurements with equalized errors, which are summed over a probe set to form the S-Score. Assuming no expression differences between chips, the S-Score follows a standard normal distribution, allowing direct tests of hypotheses to be made. Using spike-in and dilution datasets, we validated the S-Score method against comparisons of gene expression utilizing the more recently developed methods RMA, dChip, and MAS5.

**Results:**

The S-score showed excellent sensitivity and specificity in detecting low-level gene expression changes. Rank ordering of S-Score values more accurately reflected known fold-change values compared to other algorithms.

**Conclusion:**

The S-score method, utilizing probe level data directly, offers significant advantages over comparisons using only probe set expression summaries.

## Background

Affymetrix GeneChip^® ^microarrays are the most widely used and best standardized platforms for large-scale analysis of gene expression data [1,2]. Current chips are capable of measuring essentially whole genome expression values (>3 × 10^4 ^genes) simultaneously. The Affymetrix technology uses a set of probe pairs, typically 11 to 20 in number, to represent a gene [3,4]. Each probe in the probe pair is 25 bases in length. The perfect match (PM) probe corresponds exactly to the transcript of interest. The corresponding mismatch (MM) probe in the probe pair differs only in the middle (13^th^) base and is intended to measure nonspecific binding [3,4]. Prior to class comparisons, typically the signal intensities for the probe pairs in a probe set are condensed into an expression summary value, a measure representing the abundance of the corresponding gene transcript [1-3,5]. Statistical tests are then applied to these probe set expression summaries to identify which genes should be declared as differentially expressed [6].

Such an approach reflects the two central goals of statistics, estimation and inference. Although usually considered in tandem in microarray data analysis, the two steps are potentially separable [6]. The purpose of most microarray experiments is to draw inferences regarding changes in expression for a large number of genes, and estimating the level of gene expression *per se *is rarely of interest. The intermediate step of estimating expression summaries may introduce a source of variability to the analytical process, which in turn may affect error estimates used in hypothesis testing. A direct test of hypotheses using probe level data may potentially improve the accuracy of identifying differentially expressed genes. Alternatively, an increase in accuracy naturally leads to tests that offer the same statistical power using smaller sample sizes. Most algorithms have focused on improving expression summary methods, and emphasized the need for adequate numbers of replicates to ensure the accuracy of results [1,7]. This paper reports the results of our validation of the S-score algorithm, a method that offers unique advantages in the analysis of gene expression by using probe level data directly.

The S-score algorithm and software was originally designed in response to the limitations of MAS 4.0 comparison call algorithm [7,8]. It was specifically developed for comparing two hybridized GeneChips^® ^when it is of interest to identify a list of differentially expressed genes. It was developed assuming a simple error model for the expression of probe pair signals, in which the detected signal is assumed proportional to the probe pair signal for highly expressed genes, while approaching a background noise level (rather than 0) for genes with low levels of expression [8]. A similar model, with both additive and multiplicative components, has been empirically validated for cDNA microarrays [9]. For two GeneChips A and B, the error estimate for the *i*th probe pair is given by

εi=γ2(liA2+liB2)+bA2+bB2     (1)
 MathType@MTEF@5@5@+=feaafiart1ev1aaatCvAUfKttLearuWrP9MDH5MBPbIqV92AaeXatLxBI9gBaebbnrfifHhDYfgasaacH8akY=wiFfYdH8Gipec8Eeeu0xXdbba9frFj0=OqFfea0dXdd9vqai=hGuQ8kuc9pgc9s8qqaq=dirpe0xb9q8qiLsFr0=vr0=vr0dc8meaabaqaciaacaGaaeqabaqabeGadaaakeaacqaH1oqzdaWgaaWcbaGaemyAaKgabeaakiabg2da9maakaaabaGaeq4SdC2aaWbaaSqabeaacqaIYaGmaaGcdaqadaqaaiabdYgaSnaaDaaaleaacqWGPbqAcqWGbbqqaeaacqaIYaGmaaGccqGHRaWkcqWGSbaBdaqhaaWcbaGaemyAaKMaemOqaieabaGaeGOmaidaaaGccaGLOaGaayzkaaGaey4kaSIaemOyai2aa0baaSqaaiabdgeabbqaaiabikdaYaaakiabgUcaRiabdkgaInaaDaaaleaacqWGcbGqaeaacqaIYaGmaaaabeaakiaaxMaacaWLjaWaaeWaaeaacqaIXaqmaiaawIcacaGLPaaaaaa@4C9C@

where *b*_A _and *b*_B _are the background noise estimates associated with GeneChips A and B, respectively; *l*_*iA *_and *l*_*iB *_are the PM_iA_-MM_iA _and PM_iB_-MM_iB _probe pair differences for GeneChips A and B; and γ is a predefined value assumed to be constant for all GeneChips which represents the proportionality of error attributed to highly expressed genes. Therefore, γ may be thought of as the additional proportion of error attributed to *l*_*i*A _and *l*_*i*B_, which results in a larger quantity for highly abundant genes when *l*_*i*A _and *l*_*i*B _are much greater than *b*_A _and *b*_B_.

The ε_*i *_in equation (1) does not represent a rigorous statistical error estimate, but an intuitive proxy for this quantity [9]. The variance of *l*_*i*B _– *l*_*i*A_, the difference in signal intensities between GeneChips A and B, would be

Var(liA−liB)=Var(liA)+Var(liB)+bA2+bB2     (2)
 MathType@MTEF@5@5@+=feaafiart1ev1aaatCvAUfKttLearuWrP9MDH5MBPbIqV92AaeXatLxBI9gBaebbnrfifHhDYfgasaacH8akY=wiFfYdH8Gipec8Eeeu0xXdbba9frFj0=OqFfea0dXdd9vqai=hGuQ8kuc9pgc9s8qqaq=dirpe0xb9q8qiLsFr0=vr0=vr0dc8meaabaqaciaacaGaaeqabaqabeGadaaakeaacqWGwbGvcqWGHbqycqWGYbGCcqGGOaakcqWGSbaBdaWgaaWcbaGaemyAaKMaemyqaeeabeaakiabgkHiTiabdYgaSnaaBaaaleaacqWGPbqAcqWGcbGqaeqaaOGaeiykaKIaeyypa0JaemOvayLaemyyaeMaemOCaiNaeiikaGIaemiBaW2aa0baaSqaaiabdMgaPjabdgeabbqaaaaakiabcMcaPiabgUcaRiabdAfawjabdggaHjabdkhaYjabcIcaOiabdYgaSnaaDaaaleaacqWGPbqAcqWGcbGqaeaaaaGccqGGPaqkcqGHRaWkcqWGIbGydaqhaaWcbaGaemyqaeeabaGaeGOmaidaaOGaey4kaSIaemOyai2aa0baaSqaaiabdkeacbqaaiabikdaYaaakiaaxMaacaWLjaWaaeWaaeaacqaIYaGmaiaawIcacaGLPaaaaaa@5CDF@

assuming that the standard deviation of the background for GeneChips A and B is *b*_A _and *b*_B _as defined in equations (4) and (5) below. However, the variance of *l*_*i*A _and *l*_*i*B _cannot be directly estimated as there is only one observation for the probe on each chip. The equation in (1) utilizes

liA2=((PMiA−MMiA)−0)21     (3)
 MathType@MTEF@5@5@+=feaafiart1ev1aaatCvAUfKttLearuWrP9MDH5MBPbIqV92AaeXatLxBI9gBaebbnrfifHhDYfgasaacH8akY=wiFfYdH8Gipec8Eeeu0xXdbba9frFj0=OqFfea0dXdd9vqai=hGuQ8kuc9pgc9s8qqaq=dirpe0xb9q8qiLsFr0=vr0=vr0dc8meaabaqaciaacaGaaeqabaqabeGadaaakeaacqWGSbaBdaqhaaWcbaGaemyAaKMaemyqaeeabaGaeGOmaidaaOGaeyypa0ZaaSaaaeaacqGGOaakcqGGOaakcqWGqbaucqWGnbqtdaWgaaWcbaGaemyAaKMaemyqaeeabeaakiabgkHiTiabd2eanjabd2eannaaBaaaleaacqWGPbqAcqWGbbqqaeqaaOGaeiykaKIaeyOeI0IaeGimaaJaeiykaKYaaWbaaSqabeaacqaIYaGmaaaakeaacqaIXaqmaaGaaCzcaiaaxMaadaqadaqaaiabiodaZaGaayjkaiaawMcaaaaa@4882@

as a proxy variance estimate for *l*_*i*A _(and similarly for *l*_*i*B_), weighted by the factor γ.

The values of *b*_A_, *b*_B_, γ are given by

bA=SDTA=4* RawQA=4*1BGA(∑k=1BGAstdevkpixelk)*SFA     (4)
 MathType@MTEF@5@5@+=feaafiart1ev1aqatCvAUfKttLearuWrP9MDH5MBPbIqV92AaeXatLxBI9gBaebbnrfifHhDYfgasaacH8akY=wiFfYdH8Gipec8Eeeu0xXdbba9frFj0=OqFfea0dXdd9vqai=hGuQ8kuc9pgc9s8qqaq=dirpe0xb9q8qiLsFr0=vr0=vr0dc8meaabaqaciaacaGaaeqabaqabeGadaaakeaacqWGIbGydaWgaaWcbaWexLMBbXgBcf2CPn2qVrwzqf2zLnharyavP1wzZbItLDhis9wBH5gaiqaacaWFbbaabeaakiabg2da9iabbofatjabbseaejabbsfaunaaBaaaleaacqqGbbqqaeqaaOGaeyypa0JaeGinaqJaeiOkaOIaeeiiaaIaeeOuaiLaeeyyaeMaee4DaCNaeeyuae1aaSbaaSqaaiabbgeabbqabaGccqGH9aqpcqaI0aancqGGQaGkdaWcaaqaaiabigdaXaqaaiabdkeacjabdEeahnaaBaaaleaacqWGbbqqaeqaaaaakmaabmaabaWaaabCaeaadaWcaaqaaiabdohaZjabdsha0jabdsgaKjabdwgaLjabdAha2naaBaaaleaacqWGRbWAaeqaaaGcbaWaaOaaaeaacqWGWbaCcqWGPbqAcqWG4baEcqWGLbqzcqWGSbaBdaWgaaWcbaGaem4AaSgabeaaaeqaaaaaaeaacqWGRbWAcqGH9aqpcqaIXaqmaeaacqWGcbGqcqWGhbWrdaWgaaadbaGaemyqaeeabeaaa0GaeyyeIuoaaOGaayjkaiaawMcaaiabcQcaQiabdofatjabdAeagnaaBaaaleaacqWGbbqqaeqaaOGaaCzcaiaaxMaadaqadaqaaiabisda0aGaayjkaiaawMcaaaaa@76CB@

bB=SDTB=4*=RawQB=4*1BGB(∑k=1BGBstdevkpixelk)*SFB     (5)
 MathType@MTEF@5@5@+=feaafiart1ev1aqatCvAUfKttLearuWrP9MDH5MBPbIqV92AaeXatLxBI9gBaebbnrfifHhDYfgasaacH8akY=wiFfYdH8Gipec8Eeeu0xXdbba9frFj0=OqFfea0dXdd9vqai=hGuQ8kuc9pgc9s8qqaq=dirpe0xb9q8qiLsFr0=vr0=vr0dc8meaabaqaciaacaGaaeqabaqabeGadaaakeaacqWGIbGydaWgaaWcbaGaeeOqaieabeaakiabg2da9iabbofatjabbseaejabbsfaunaaBaaaleaacqqGcbGqaeqaaOGaeyypa0JaeGinaqJaeiOkaOIaeyypa0JaeeOuaiLaeeyyaeMaee4DaCNaeeyuae1aaSbaaSqaaiabbkeacbqabaGccqGH9aqpcqaI0aancqGGQaGkdaWcaaqaaiabigdaXaqaaiabdkeacjabdEeahnaaBaaaleaacqWGcbGqaeqaaaaakmaabmaabaWaaabCaeaadaWcaaqaaiabdohaZjabdsha0jabdsgaKjabdwgaLjabdAha2naaBaaaleaacqWGRbWAaeqaaaGcbaWaaOaaaeaacqWGWbaCcqWGPbqAcqWG4baEcqWGLbqzcqWGSbaBdaWgaaWcbaGaem4AaSgabeaaaeqaaaaaaeaacqWGRbWAcqGH9aqpcqaIXaqmaeaacqWGcbGqcqWGhbWrdaWgaaadbaGaemOqaieabeaaa0GaeyyeIuoaaOGaayjkaiaawMcaaiabcQcaQiabdofatjabdAeagnaaBaaaleaacqWGcbGqaeqaaOGaaCzcaiaaxMaadaqadaqaaiabiwda1aGaayjkaiaawMcaaaaa@6A2C@

γ = 0.1     (6)

where SDT_A _and SDT_B _are the Statistical (or Standard) Difference Threshold (SDT) values of GeneChip A and B, respectively. RawQ is an estimate of the background noise, where BG is the number of probes used in the background estimate; stdev_k _and pixel_k _are the standard deviation and number of pixels for the *k*th probe; and the Scale Factor (SF) is used to scale each of the intensities on the chip to a specified target background value [10]. The values of RawQ and SF are available from the Affymetrix GeneChip Operating Software (GCOS). The value of γ was chosen as indicated in equation (6) so that the scale of the S-scores does not depend on the expression levels of a gene. This is consistent with previous work showing that the additive component of the error model (1) varies from array to array (and so is derived from the background fluctuation level for each array), while the fractional multiplicative error is fairly constant [9].

These probe pair level error estimates are then used in the calculation of a new measure of relative change in gene expression, called the significance score or S-score. A relative change in probe pair intensities is calculated that converts the probe pair signal differences into multiple measurements with equalized errors. These relative changes are then summed to form the S-score, which is a single measure of the significance of change for the gene in question. For probe set *j*, the S-score is calculated as

Sj=∑i=1NjliB−liAαεiNj     (7)
 MathType@MTEF@5@5@+=feaafiart1ev1aqatCvAUfKttLearuWrP9MDH5MBPbIqV92AaeXatLxBI9gBaebbnrfifHhDYfgasaacH8akY=wiFfYdH8Gipec8Eeeu0xXdbba9frFj0=OqFfea0dXdd9vqai=hGuQ8kuc9pgc9s8qqaq=dirpe0xb9q8qiLsFr0=vr0=vr0dc8meaabaqaciaacaGaaeqabaqabeGadaaakeaacqWGtbWudaWgaaWcbaGaemOAaOgabeaakiabg2da9maaqahabaWaaSaaaeaacqWGSbaBdaWgaaWcbaGaemyAaKMaemOqaieabeaakiabgkHiTiabdYgaSnaaBaaaleaacqWGPbqAcqWGbbqqaeqaaaGcbaGaeqySdeMaeqyTdu2aaSbaaSqaaiabdMgaPbqabaGcdaGcaaqaaiabd6eaonaaBaaaleaacqWGQbGAaeqaaaqabaaaaaqaaiabdMgaPjabg2da9iabigdaXaqaaiabd6eaonaaBaaameaacqWGQbGAaeqaaaqdcqGHris5aOGaaCzcaiaaxMaadaqadaqaaiabiEda3aGaayjkaiaawMcaaaaa@4D0D@

where *l*_*i*A_, *l*_*i*B_, and ε_*i *_are as in equation (1); *N*_*j *_is the number of probe pairs within the probe set; and α is a normalization factor that corrects for the effect of correlation among probe pair signals. The value of α was chosen for an individual chip so that the variance of S-score values on an array is 1 when outliers are excluded. Under these conditions, for non-differentially expressed genes, the S-score follows a standard normal distribution [8,9]. Thus, p-values are readily calculated and used to determine the significance of change in gene expression. The S-score method thereby eliminates the need for estimation of probe set expression summaries, simplifying the analytical process. S-scores, by virtue of their direct comparison of individual probe-pair data, provide comparison of the expression change between two chips. This allows at least inferential statistics on experiments with limited numbers of microarrays [8].

The S-score has been used in selecting differentially expressed genes in peer-reviewed, published studies [11-13], but has not yet achieved widespread use despite its attractive features. This may be due to concerns that the initial validation studies of the S-score algorithm utilized data sets for which the identification of genes that were differentially expressed were not known, and therefore may be considered inadequate by today's standards. Therefore, we performed an additional empirical validation study of the S-score algorithm against comparisons utilizing more recently developed methods – RMA, MAS5 and dChip – using data sets in which each probe set was known to be either differentially or non-differentially expressed. Such an analysis would also determine whether hypothesis testing using probe level data directly offers advantages over testing using expression summaries.

## Results

For the quality control measures, quantile-quantile plots for the Dilution dataset showed that the assumption of a single distribution is reasonable (Additional File 4). The Latin Square dataset showed problems with linearity, which was especially notable for chips 92562, 92563, and 92564 where the R^2 ^values were less than 0.15 (Additional File 5). Analyses were repeated after excluding these three chips (Additional File 6, Tables 13 through 20). The impact of this departure on the analysis is discussed below.

### Dilution dataset

**Figure 1:**
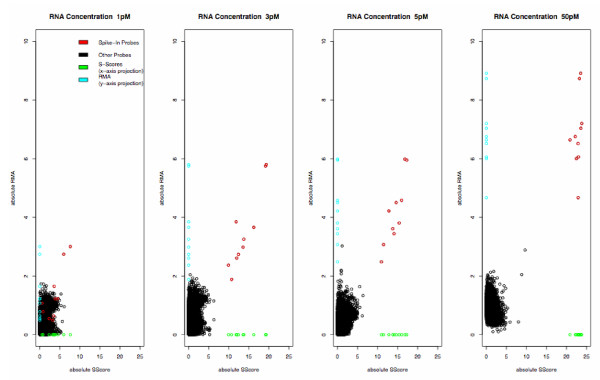
**Comparison of S-Score and RMA**. Plot of absolute value of S-Score vs absolute value of difference in RMA expression summaries, comparing the specified concentration to the baseline chip. X- and Y-axis projections are added to show separation of spike-in probes more clearly.

**Figure 2:**
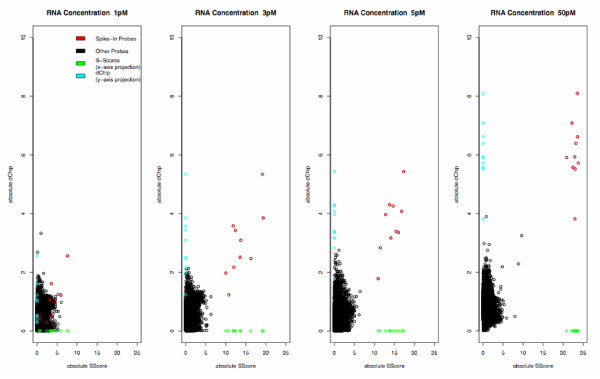
**Comparison of S-Score and dChip**. Plot of absolute value of S-Score vs absolute value of difference in base 2 logarithm of dChip model-based expression index, comparing the specified concentration to the baseline chip. X- and Y-axis projections are added to show separation of spike-in probes more clearly.

**Figure 3:**
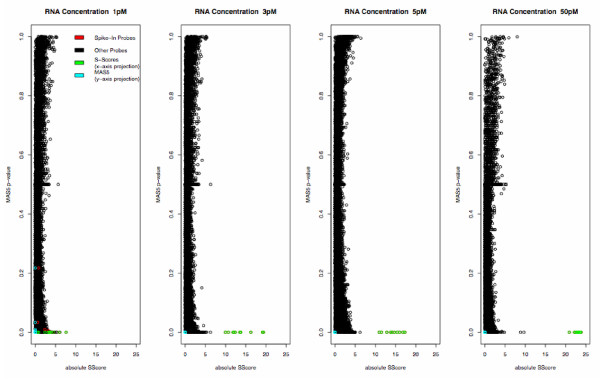
**Comparison of S-Score and MAS5**. Plot of absolute value of S-Score vs MAS5 p-values, comparing the specified concentration to the baseline chip. MAS5 p-values were transformed so that significantly up- and down-regulated genes will have p-values approaching 0. X- and Y-axis projections are added to show separation of spike-in probes more clearly.

Typical plots of S-score values against other algorithms for representative concentration levels are shown in Figures 1-3. (A full set of plots for all but the highest concentration is provided in Additional Files 1-3. The 150 pM condition was omitted for reasons of space.) The S-score values clearly separated the spike-in clones from other probe sets at concentrations of 3 pM and greater, with some loss of accuracy at lower concentrations. RMA expression summary values also separated the spike-in clones from the remaining probe sets, although this did not occur completely until concentrations of 12.5 pM and greater. The MBEI values produced by dChip did not provide total separation at any concentration, although definite improvement was noted with concentrations of 5 pM and greater. Similarly, MAS5 p-values did not provide total separation at any concentration.

### Latin square dataset

For each GeneChip analyzed from the Latin Square dataset, observed ranks of the spike-in clone probe sets for each algorithm were examined in comparison to their true underlying rank using chip 92561 as the reference (Table 1). Similar results were obtained when other chips were used as the reference (Additional File 6, Tables 3 through 12). Ideally, the observed rank should equal the true underlying rank. Therefore, the proportion of spike-in clones with ranks less than or equal to 11 should be 1.0. Further, it should be noted that as the observed rank for the spike-in clones falls, it becomes more likely that the associated probe set will fail to be identified as differentially expressed, and hence will be missed as an important gene (probe set) for further study (i.e. sensitivity decreases). The MAS5 algorithm had the highest proportion of clones in the top 11 (Table 2), though it had difficulty in separating the clones from each other despite clear differences in fold-change (Table 1). Compared to RMA and dChip, the observed ranks for the S-score are generally much closer to the true underlying ranks, and the proportion of clones in the top 11 is higher (Table 2). These differences were statistically significant between the S-score and RMA (χ^2^(1) = 17.88, p < 0.001) and between the S-score and dChip (χ^2^(1) = 21.33, p < 0.001). The differences between the S-score and MAS5 were not statistically significant (χ^2^(1) = 0.40, p > 0.52). Analyses conducted using other chips as the baseline exhibited similar trends, although the results were not always statistically significant. Analyses conducted after excluding arrays 92562, 92563, and 92564 showed the performance of the S-Score, RMA, and MAS5 to be comparable (χ^2^(1) > 0.51 for all comparisons of the S-Score versus RMA and χ^2^(1) > 0.26 for all comparisons of the S-Score versus MAS5).

## Discussion

This study validates the S-score using standardized datasets that were unavailable at the time the algorithm was developed. In their original paper, Zhang and colleagues provided initial validation of the S-score using three different methods [8]. First, the S-score values were clearly reproducible when comparing dissimilar brain regions, where many gene expression differences would be expected (R = 0.75). This was not the case when comparing similar brain regions, where few expression differences would be expected (R = 0.17). Second, the S-score values were found to be more consistent than MAS4 in labeling expression differences between dissimilar brain regions, without loss of sensitivity. Third, clusters generated using the S-score were much tighter than those generated using the logarithm of the fold-change ratio, ln(Fc), with an average R of 0.80 and 0.52 respectively. Later work yielded results similar to the initial validation, finding the S-score values highly reproducible between dissimilar brain regions (R = 0.65) but not between similar brain regions (R = 0.00002) [7]. Finally, a reanalysis of a previous study using the S-score generally confirmed the prior results, but also revealed a number of genes with significant, reproducible changes that were not identified in the original analysis [14].

However, since all of these validation studies involved experimental samples, the true gene expression changes were unknown. By using datasets in which individual probes are spiked in at known concentrations, the accuracy of the algorithm can be externally validated by independent means. Using two widely available spike-in datasets, the S-score compares very favorably to the more recently developed algorithms available in RMA, dChip, and MAS5 in detecting differential gene expression.

The Dilution dataset assesses the sensitivity and specificity of each algorithm at various concentrations, and allows a determination of the limits of detection. The S-score exhibited an excellent combination of sensitivity and specificity in the detection of differentially expressed genes, clearly separating the spike-in clones from the other probe sets except at the lowest concentrations. The S-score and RMA outperformed both dChip and MAS5, with the S-score capable of separating the spike-ins from other probe sets at slightly lower concentration than RMA.

The Latin Square dataset assesses the performance of each algorithm under more realistic conditions, where expression differences vary by gene. In such situations, investigators will often be interested in those genes showing the most significant changes between experimental and control conditions. This is typically accomplished by ranking genes by increasing order of significance, and selecting the top *M *ranked genes for further study. Thus, it is critical for an algorithm to assign observed ranks that are similar to expected ranks that would be obtained using the known fold-change in gene expression; otherwise, genes that play a critical role in the difference between the experimental and control condition might be overlooked. The arrangement of spike-in concentrations in the Latin Square dataset allows expected ranks to be calculated based on the true fold-change and compared to the observed ranks generated by the different algorithms. Again, the S-score compared favorably to the other three algorithms. MAS5 did perform slightly better, with a higher proportion of spike-in genes ranked in the top 11, though the difference was not statistically significant. It is also concerning that the MAS5 p-values were unable to differentiate among the spike-in genes despite clear differences in fold-change. This is particularly critical if resources permit follow-up of only a limited subset of genes; in such situations, the MAS5 p-values would provide little help in choosing from the list of genes to explore. The S-score had significantly better performance than RMA or dChip, with a greater proportion of spike-in genes ranked in the top 11 than the proportion obtained using either of the other two programs. After excluding three arrays of potentially poor quality, RMA was able to equal the performance of the S-Score on most chips and slightly outperform the S-Score on a small number of chips, although the difference was not significant. MAS5 continued to detect a larger number of spike-in probes, though again the difference was not significant.

Some limitations of this investigation must be noted. The analytical methods, particularly for RMA and dChip, were unusual in that replicate experiments were not used. Currently, the S-score method and its software implementation allow only the comparison of two chips at a time. Thus, the datasets were limited to one chip for each experiment so that the conditions would be similar for all four algorithms. Clearly, replication is necessary to assess biological variation. When multiple chips per condition are available, the utility of the other algorithms – RMA, dChip, and MAS5 – in detecting differentially expressed genes has been well documented. However, this study provides evidence that additional refinement might be achieved using methods similar to the S-score, which perform tests of hypotheses based on probe level data rather than expression summaries. Further work is clearly needed in extending the S-score method to allow comparison of multiple chips simultaneously, as the biological significance of the gene expression changes can only be addressed using replicate experiments [8]. A limitation of using the S-Score for a two-chip comparison is that it is possible that a large observed S-score might be indicative of a defect in the chip (or other unexplained factors) rather than a biologically significant change [8]. Such an occurrence would not be a problem for the current study, where the biological truth is known, but would be of concern in studies involving only experimental data sets. Nevertheless, the results of this study provide excellent justification for further development of the S-score method. Such extensions of the S-Score are currently being developed, using a mixed-effects approach to model the probe level data for multiple GeneChips.

Another limitation of this study relates to the datasets examined. Many standard quality control measures for microarray data could not be applied to these datasets. Thus, while the data used in this study are generally believed to be of good quality, this is difficult to verify. This may be a particular issue for the Latin Square dataset, where several probes had markedly different values between expected and observed ranks. Examination of GeneChip level plots of concentration of spike-in by expression revealed why problems in detecting differential expression among specific comparisons may be difficult. That is, for some probe sets, the absolute expression change is likely too small to be detected, even though the fold-change is great (e.g. a change from 0.5 pM to 1.5 pM). For other probe sets, the degree of true fold-change is likely too small to be detected (e.g. a change of 1.3-fold from 25 pM to 35.7 pM). However, there remain a small number of probes where the known and calculated ranks are markedly different without an obvious explanation. These problems were encountered with all four algorithms, and were most notable with chips showing a poor degree of linearity when examining concentration of spike-in and expression for the probe sets. It is unknown if these differences in the ranks might be due to poor chip quality, hybridization conditions under which these chips were run, or scanning issues.

## Conclusion

In summary, the S-score algorithm utilizes a novel approach to detecting differential gene expression, basing tests of hypotheses on probe level data rather than expression summaries. Results indicate that such a method performs very favorably compared to other currently available methods using a standardized dataset. Further research is needed to confirm these results and fully explore the gains that may be achieved using probe level data directly; some of these goals may be realized by current efforts to refine the S-score method. The analysis of gene expression data is a complex and evolving field, and the S-score algorithm offers distinct advantages that make it an attractive option for analysis of oligonucleotide microarray experiments.

## Methods

### Data

The data for this study were drawn from two datasets (i.e., Dilution and Latin Square) created by Gene Logic, Inc. using the human U95 GeneChip™ [14]. Each dataset consists of a series of *.CEL files, with one file for each chip hybridized. For both datasets, a common complex cRNA derived from an acute myeloid leukemia (AML) tumor cell line was hybridization to each chip. Prior to hybridization, clones from different regions of 4 bacterial genes (BioB, BioC, BioD, and Dap) and of 1 phagemid gene (Cre) were spiked into the sample at known concentrations. For the Dilution data set, 10 different clones were spiked into the hybridization cocktail at the same concentration on each array. The concentrations ranged from 0.5 to 150 pM, with 1 to 3 replicates at each level (Additional File 6,  Table 1). For the Latin Square data set, 11 clones were spiked into the hybridization cocktail using a different concentration arranged in a Latin Square design. The concentrations ranged from 0.5 to 100 pM, with 2 to 3 replicates at each level (Additional File 6).

### Statistical methods

Since we were comparing two GeneChips at a time, it was necessary to identify a baseline GeneChip to which all other chips were compared. For the Dilution dataset, the 0 pM concentration (chip 92466) was used as a baseline to which the remaining GeneChips were compared. For the Latin Square data set, the BioB-5 0.5 pM concentration (chip 92561) was used as a baseline for the initial analysis. Since the choice of baseline chip for this data set is arbitrary, analyses were repeated using each chip in turn as the baseline chip. For attaining optimal sensitivity and specificity, comparisons using each algorithm (S-Score, RMA, dChip, and MAS5) should identify all 10 (Dilution data) or 11 (Latin Square data) spiked probe sets as differentially expressed. Identification of fewer probe sets among these 10 or 11 would be false negative findings, while identification of probe sets in addition to these would be false positive findings. Therefore, using this information, sensitivity and specificity of comparisons made with each algorithm can be estimated.

Prior to analysis, a quality assessment was performed on each chip. Because of the nature of the spike-in experiments, many tests for quality control, such as RNA degradation, could not be performed. Assessment of linearity and lack of fit, another quality control measure, also could not be performed due to lack of replicates. The Dilution dataset did not have multiple concentrations on a single chip, and the Latin Square dataset did not have multiple probes at the same concentration on each chip. For the Dilution dataset, the intensities of all probe sets at a fixed concentration level should be similar under the assumption of linearity. Quantile-quantile plots of the MAS5 intensity values were used to test the assumption that the intensities were from a single distribution with a common mean. For the Latin Square dataset, plots of probe set concentration versus the MAS5 intensity value were generated for each chip. Visual inspection of linearity within a chip was supplemented with calculation of the R^2 ^value of the linear regression equation.

In comparing the four methods using the Dilution data set, the *.CEL files were read into the appropriate program for analysis and commonly used measures for declaring genes differentially expressed were calculated. That is, RMA expression summaries were determined using the *rma *function in version 1.6.7 of the *affy *package [1] and R version 2.1.0 [15] The expression change was calculated as the absolute difference in expression summaries between the chip of interest and the baseline chip. MAS5 expression change p-values were calculated between the chip of interest and the baseline chip using the GCOS version 1.1.1 [16]. As described in the Affymetrix GCOS manual, p-values near 0 or 1 are indicative of differential expression, while p-values near 0.5 are indicative of no differential expression. Thus, we transformed the Affymetrix p-values *p *to a common scale p* ranging from 0 to 1, with low values indicating significant change:

p*={2*p          if p < 0.52*(1−p)   if p ≥ 0.5     (8)
 MathType@MTEF@5@5@+=feaafiart1ev1aqatCvAUfKttLearuWrP9MDH5MBPbIqV92AaeXatLxBI9gBaebbnrfifHhDYfgasaacH8akY=wiFfYdH8Gipec8Eeeu0xXdbba9frFj0=OqFfea0dXdd9vqai=hGuQ8kuc9pgc9s8qqaq=dirpe0xb9q8qiLsFr0=vr0=vr0dc8meaabaqaciaacaGaaeqabaqabeGadaaakeaacqWGWbaCdaahaaWcbeqaaiabcQcaQaaakiabg2da9maaceaaeaqabeaacqaIYaGmcqGGQaGkcqWGWbaCcaaMe8UaaGjbVlaaysW7caaMe8UaaGjbVlaaysW7caaMe8UaaGjbVlaaysW7caaMe8UaeeyAaKMaeeOzayMaeeiiaaIaeeiCaaNaeeiiaaIaeeipaWJaeeiiaaIaeeimaaJaeeOla4IaeeynaudabaGaeGOmaiJaeiOkaOIaeiikaGIaeGymaeJaeyOeI0IaemiCaaNaeiykaKIaaGjbVlaaysW7caaMe8UaeeyAaKMaeeOzayMaeeiiaaIaeeiCaaNaeeiiaaIaeyyzImRaeeiiaaIaeeimaaJaeeOla4IaeeynaudaaiaawUhaaiaaxMaacaWLjaWaaeWaaeaacqaI4aaoaiaawIcacaGLPaaaaaa@684C@

For the dChip method, the data were transformed using the base 2 logarithm. The Li & Wong model based expression index (MBEI) was then calculated using a PM only model in dChip version 1.3 [17]. The expression change was calculated as the absolute difference in the MBEI between the chip of interest and the baseline chip. For the S-score method, S-scores were determined using the *SScoreBatch *function in version 1.1.1 of the *SScore *package [18] in R version 2.1.0. Values for the Scale Factor (SF) parameter and RawQ were obtained from the GCOS 1.1.1 output, and the Statistical Difference Threshold (SDT) parameter was calculated as 4 times RawQ times the Scale Factor. The S-scores were used directly as a measure of expression change. Plots of the S-scores versus each of the other algorithms were used to assess the comparative ability of each algorithm to clearly separate the spike-in clones from the remaining probe sets.

Due to the varying concentration of spike transcripts in the Latin Square experiment, a different procedure for comparing the four algorithms was conducted. As described with the Dilution dataset, the *.CEL files were read into the appropriate program and commonly used measures for declaring genes differentially expressed were calculated. Probe sets were then rank ordered based on the results provided by each algorithm. Calculation of ranks was carried out using JMP version 5.1 [19]. Rankings from each algorithm were compared to the true underlying fold-change values of the spike-in clones. The true underlying fold-change ranks were determined using the concentration of the spike-in clones (Additional File 6, Table 2) for the two chip comparisons. A comparative method would have optimal performance if all of the spike-in clones were ranked among the top 11 genes identified as differentially expressed. Therefore, the proportion of spike-ins ranked less than or equal to 11 was calculated, and the Cochran-Mantel-Hanzel test used to compare these proportions across all chips.

### Implementation

The S-score algorithm is available through Bioconductor[20]and is currently implemented in version 1.1.1 of the *SScore *package [18], which runs in R version 1.8 or later. An implementation using Borland Delphi version 5 and compiled as a stand-alone program for the Windows operating system is also available [21].

## Authors' contributions

RK conceived the study, performed the statistical analysis and drafted the manuscript. KA and MM participated in manuscript preparation. All authors read and approved the final manuscript.

## Supplementary Material

Additional File 4 **Quantile-quantile plots of intensity data for the Dilution dataset**. Click here for file

Additional File 5 **Linearity plots for the Latin Square dataset**. Click here for file

Additional File 6 **Supplementary Tables 1-20**. Click here for file

Additional File 1 **Comparison of S-Score and RMA**. Plot of absolute value of S-Score vs absolute value of difference in RMA expression summaries, comparing the specified concentration to the baseline chip. X- and Y-axis projections are added to show separation of spike-in probes more clearly. Click here for file

Additional File 2 **Comparison of S-Score and dChip**. Plot of absolute value of S-Score vs absolute value of difference in base 2 logarithm of dChip model-based expression index, comparing the specified concentration to the baseline chip. X- and Y-axis projections are added to show separation of spike-in probes more clearly. Click here for file

Additional File 3 **Comparison of S-Score and MAS5**. Plot of absolute value of S-Score vs MAS5 p-values, comparing the specified concentration to the baseline chip. MAS5 p-values were transformed so that significantly up- and down-regulated genes will have p-values approaching 0. X- and Y-axis projections are added to show separation of spike-in probes more clearly. Click here for file

## Figures and Tables

**Table 1:** Observed and expected ranks. Observed and expected ranks from the Latin Square dataset for each of the four comparative methods, with linear correlation (R^2^) of MAS5 intensity vs concentration as quality control data.

Chip	**92562 (R^2 ^= 0.042)**					**92563 (R^2 ^= 0.123)**				
Probe Set	**Rank**	**Observed Rank**				Rank	Observed Rank			

		**S-Score**	**RMA**	**dChip**	**MAS5**		S-Score	RMA	dChip	MAS5

BioB-5	**4**	**5042**	**7182**	**88**	**491**	7	11826	9395	125	539
BioB-M	**8**	**20**	**5898**	**93**	**516**	8	9	11023	136	61
BioB-3	**7**	**1966**	**3490**	**74**	**516**	9	7	11810	145	3
BioC-5	**9**	**391**	**1336**	**53**	**110**	1	3	2	2	1
BioC-3	**1**	**5**	**5**	**11**	**1**	2	4	3	4	1
BioDn-3	**6**	**1**	**3**	**5**	**1**	9	5	12560	179	1
DapX-5	**9**	**9652**	**1526**	**57**	**450**	4	2	4	3	1
DapX-M	**6**	**3**	**1**	**1**	**1**	9	64	9497	132	96
DapX-3	**5**	**2**	**2**	**2**	**1**	6	6	12443	155	1
CreX-5	**2**	**7018**	**1795**	**66**	**372**	3	1	1	1	1
CreX-3	**3**	**501**	**7575**	**95**	**516**	5	1080	4811	92	251
										
Chip	92564 (R^2 ^= 0.047)					**92558 (R^2 ^= 0.234)**				

Probe Set	Rank	Observed Rank				**Rank**	**Observed Rank**			

		S-Score	RMA	dChip	MAS5		**S-Score**	**RMA**	**dChip**	**MAS5**

BioB-5	8	9628	4393	96	513	**6**	**8**	**8**	**8**	**1**
BioB-M	10	5	213	147	1	**2**	**3**	**3**	**2**	**1**
BioB-3	9	6	47	140	1	**1**	**2**	**2**	**3**	**1**
BioC-5	4	2	12626	1	1	**9**	**5**	**5**	**6**	**1**
BioC-3	5	4	2119	112	1	**10**	**6**	**6**	**7**	**1**
BioDn-3	3	8	359	138	1	**1**	**1**	**4**	**4**	**1**
DapX-5	2	1	12625	2	1	**5**	**12**	**13**	**30**	**1**
DapX-M	1	1656	7093	105	513	**7**	**10**	**7**	**9**	**1**
DapX-3	6	3	1233	122	1	**3**	**7**	**9**	**13**	**1**
CreX-5	1	7	320	145	1	**8**	**4**	**1**	**1**	**1**
CreX-3	7	22	11569	51	16	**4**	**9**	**11**	**18**	**1**
										
Chip	**92559 (R^2 ^= 0.302)**					92560 (R^2 ^= 0.745)				

Probe Set	**Rank**	**Observed Rank**				Rank	Observed Rank			

		**S-Score**	**RMA**	**dChip**	**MAS5**		S-Score	RMA	dChip	MAS5

BioB-5	**6**	**10**	**8**	**9**	**1**	2	5	4	4	1
BioB-M	**3**	**1**	**3**	**3**	**1**	7	9	189	74	1
BioB-3	**4**	**2**	**4**	**4**	**1**	3	1	2	2	1
BioC-5	**8**	**5**	**6**	**7**	**1**	6	4	5	5	1
BioC-3	**9**	**7**	**7**	**8**	**1**	9	6	6	6	1
BioDn-3	**9**	**9**	**16**	**73**	**1**	8	10	881	87	1
DapX-5	**7**	**4**	**2**	**2**	**1**	6	3	3	3	1
DapX-M	**5**	**6**	**5**	**6**	**1**	4	265	7339	115	130
DapX-3	**1**	**11**	**12**	**17**	**1**	1	7	7	9	1
CreX-5	**9**	**3**	**1**	**1**	**1**	9	2	1	1	1
CreX-3	**2**	**8**	**9**	**11**	**1**	5	670	2511	95	126
										
Chip	92554 (R^2 ^= 0.874)					**92555 (R^2 ^= 0.668)**				

Probe Set	Rank	Observed Rank				**Rank**	**Observed Rank**			

		S-Score	RMA	dChip	MAS5		**S-Score**	**RMA**	**dChip**	**MAS5**

BioB-5	3	1	1	1	1	**1**	**2**	**1**	**1**	**1**
BioB-M	6	4	5	6	1	**5**	**7**	**10**	**11**	**1**
BioB-3	5	3	3	4	1	**4**	**5**	**6**	**6**	**1**
BioC-5	1	8	12	37	1	**2**	**10**	**7**	**7**	**1**
BioC-3	2	7	8	9	1	**3**	**11**	**8**	**8**	**1**
BioDn-3	7	6	14	27	1	**1**	**1**	**3**	**2**	**1**
DapX-5	4	10	6	13	1	**3**	**9**	**9835**	**127**	**1**
DapX-M	4	2	2	2	1	**2**	**3**	**2**	**3**	**1**
DapX-3	9	5	4	5	1	**6**	**6**	**5**	**5**	**1**
CreX-5	4	11	7	14	1	**6**	**4**	**4**	**4**	**1**
CreX-3	8	19	178	64	4	**4**	**8**	**9089**	**122**	**1**
										
Chip	**92556 (R^2 ^= 0.748)**					92557 (R^2 ^= 0.756)				

Probe Set	**Rank**	**Observed Rank**				Rank	Observed Rank			

		**S-Score**	**RMA**	**dChip**	**MAS5**		S-Score	RMA	dChip	MAS5

BioB-5	**1**	**3**	**1**	**1**	**1**	1	2	2	2	1
BioB-M	**3**	**2**	**3**	**2**	**1**	4	4	3	1	1
BioB-3	**8**	**7**	**22**	**37**	**1**	2	3	1	3	1
BioC-5	**4**	**9**	**19**	**29**	**1**	3	14	373	54	1
BioC-3	**4**	**8**	**6**	**11**	**1**	9	5	300	56	1
BioDn-3	**4**	**1**	**4**	**4**	**1**	5	1	4	4	1
DapX-5	**8**	**6**	**8**	**9**	**1**	10	9	9	10	1
DapX-M	**2**	**4**	**2**	**3**	**1**	11	10	87	78	1
DapX-3	**5**	**14**	**11**	**23**	**1**	6	7	7596	107	1
CreX-5	**7**	**5**	**5**	**7**	**1**	8	8	16	19	1
CreX-3	**6**	**10**	**7**	**12**	**1**	7	6	1151	72	1

**Table 2:** Number and proportion of spike-in clones detected using chip 92561 as baseline

	**Clones Detected**			
**GeneChip Array**	**S-Score**	**RMA**	**dChip**	**MAS5**

92562	4 (0.36)	4 (0.36)	4 (0.36)	4 (0.36)
92563	8 (0.72)	4 (0.36)	4 (0.36)	7 (0.64)
92564	8 (0.72)	0 (0.00)	2 (0.18)	8 (0.72)
92558	10 (0.90)	10 (0.90)	8 (0.72)	11 (1.00)
92559	11 (1.00)	9 (0.81)	9 (0.81)	11 (1.00)
92560	9 (0.81)	7 (0.63)	7 (0.63)	9 (0.81)
92554	10 (0.90)	8 (0.72)	6 (0.54)	11 (1.00)
92555	11 (1.00)	9 (0.81)	9 (0.81)	11 (1.00)
92556	10 (0.90)	9 (0.81)	7 (0.63)	11 (1.00)
92557	10 (0.90)	5 (0.45)	5 (0.45)	11 (1.00)

Comparison of S-Score vs. RMA, p < 0.001; vs. dChip, p < 0.001; vs. MAS5, p = 0.40
