# Supplementary figures and images for: Empirical validation of the S-Score algorithm in the analysis of gene expression data
Source: BMC Bioinformatics. 2006 Mar 17;7:154. doi: 10.1186/1471-2105-7-154 (PMC1550434; doi:10.1186/1471-2105-7-154)

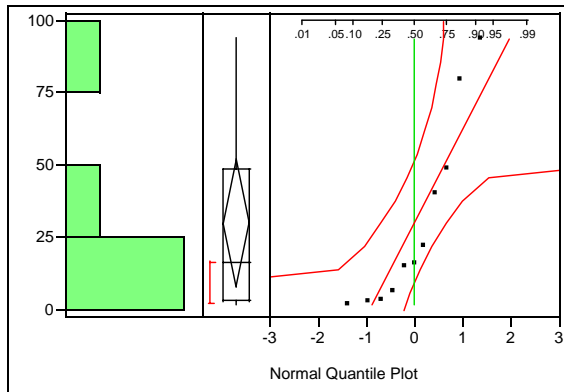

Chip 92491

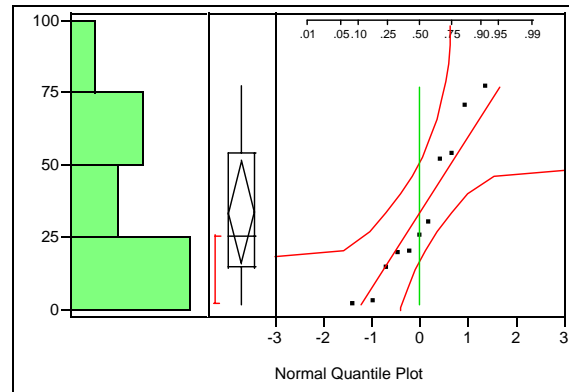

Chip 92494

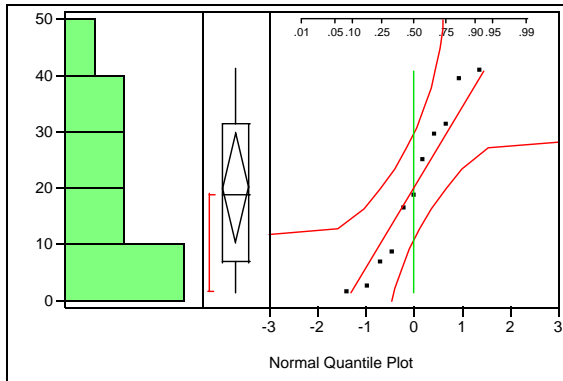

Chip 92492

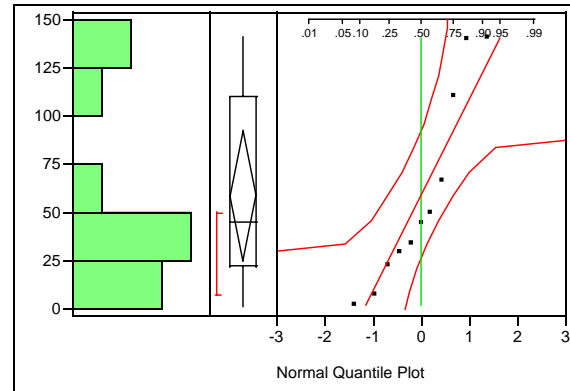

Chip 92495

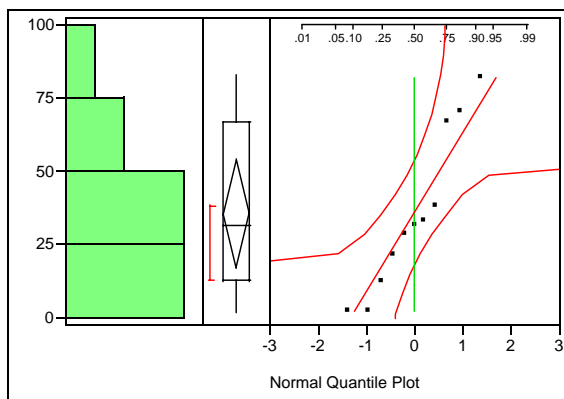

Chip 92493

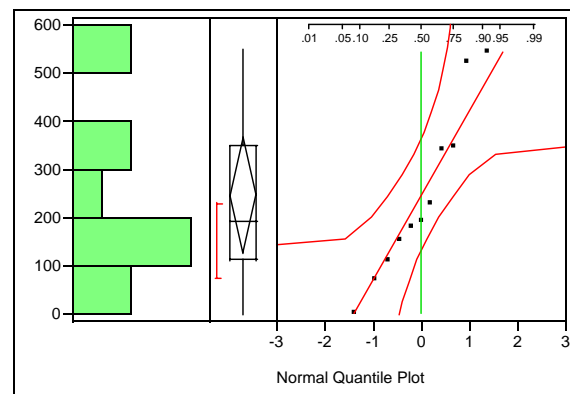

Chip 92496

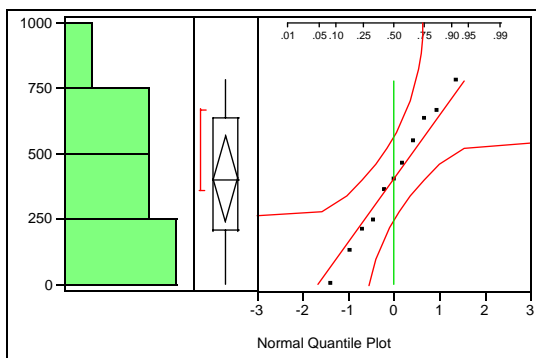

Chip 92453

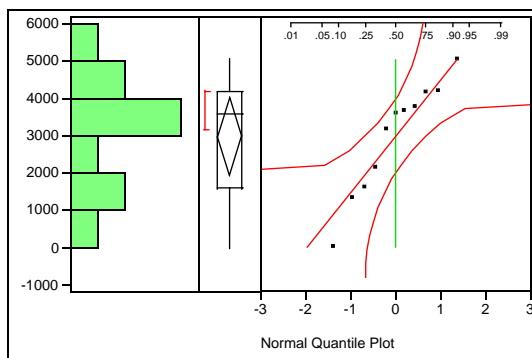

Chip 92460

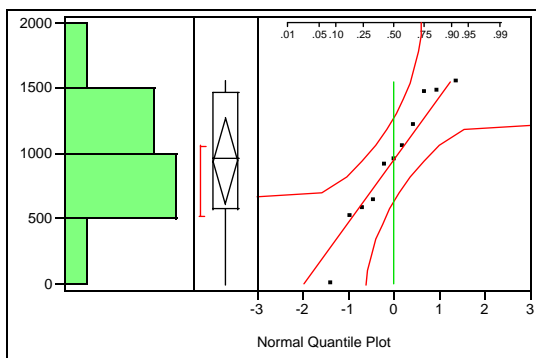

Chip 92454

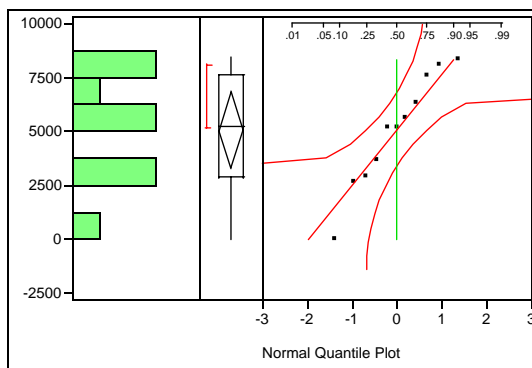

Chip 92462

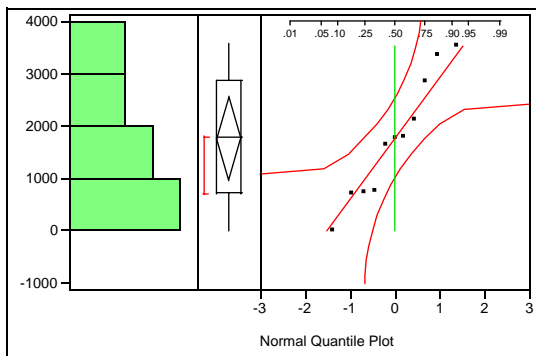

Chip 92456

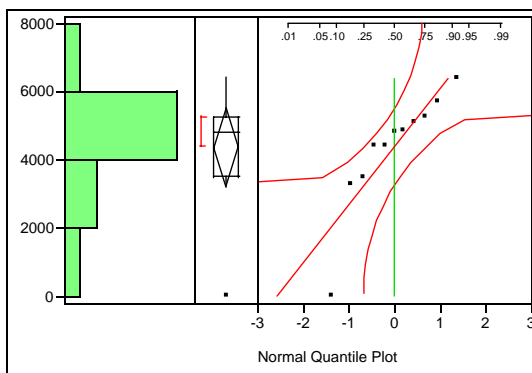

Chip 92464

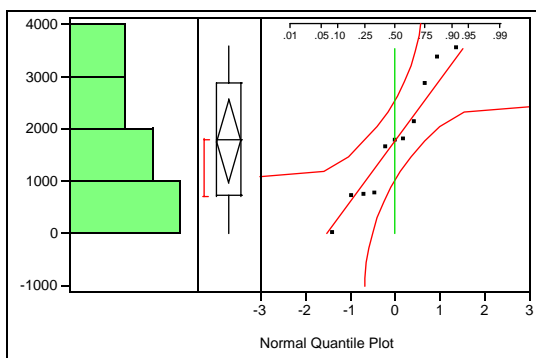

Chip 92458

Supplement: Additional File 4 — Quantile-quantile plots of intensity data for the Dilution dataset. [file 1471-2105-7-154-S4.pdf]

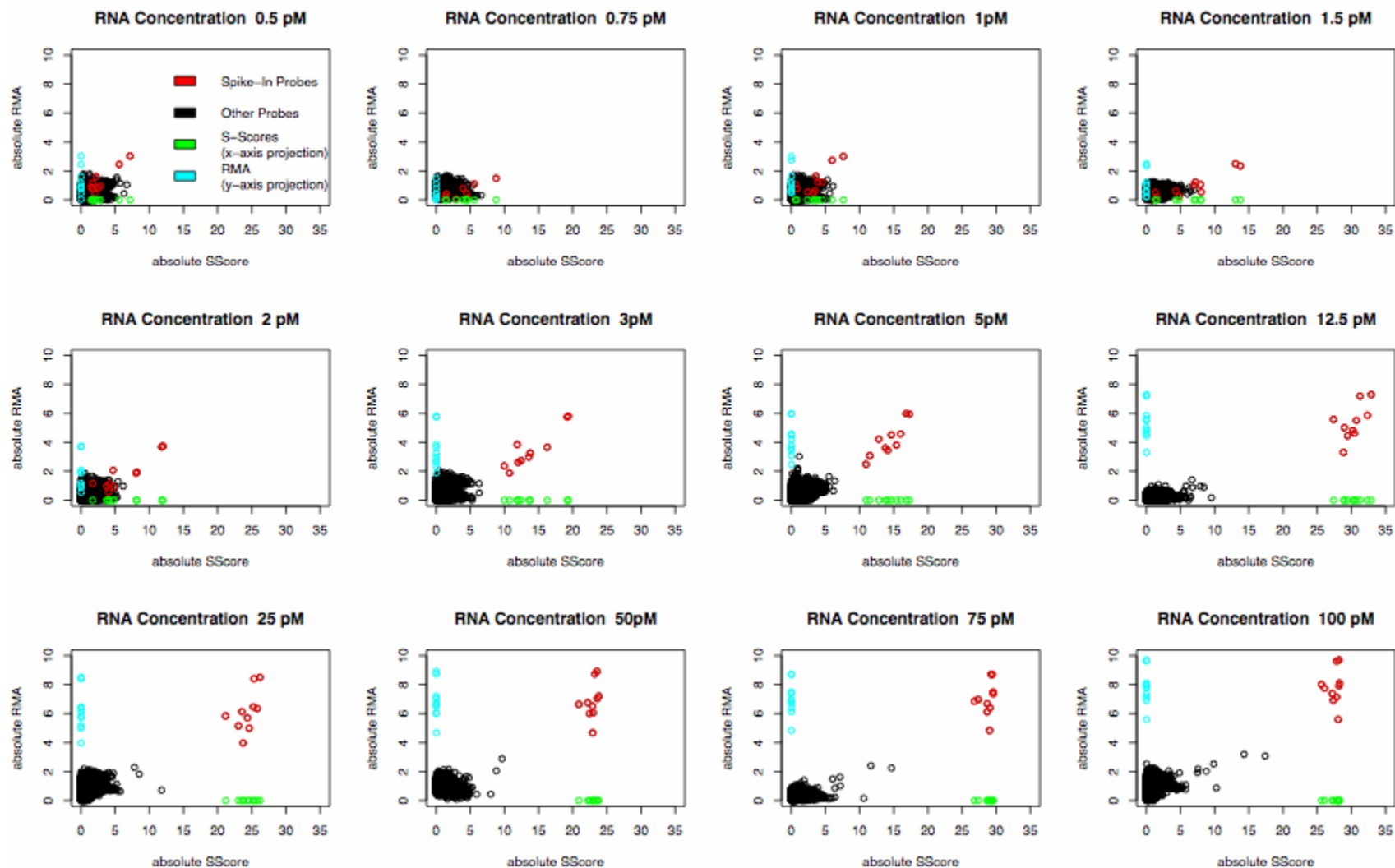

Supplement: Additional File 1 — Comparison of S-Score and RMA. Plot of absolute value of S-Score vs absolute value of difference in RMA expression summaries, comparing the specified concentration to the baseline chip. X- and Y-axis projections are added to show separation of spike-in probes more clearly. [file 1471-2105-7-154-S1.pdf]

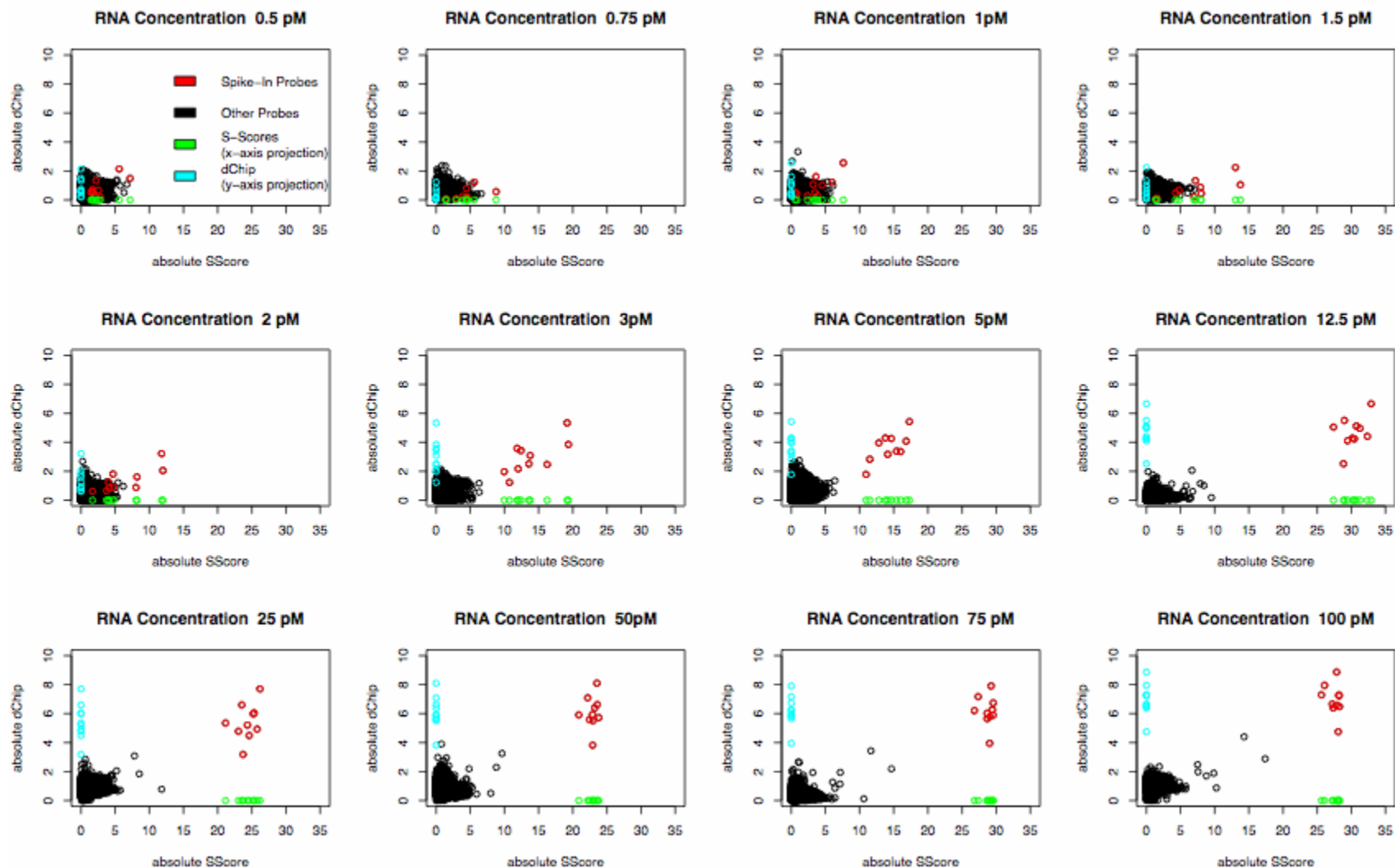

Supplement: Additional File 2 — Comparison of S-Score and dChip. Plot of absolute value of S-Score vs absolute value of difference in base 2 logarithm of dChip model-based expression index, comparing the specified concentration to the baseline chip. X- and Y-axis projections are added to show separation of spike-in probes more clearly. [file 1471-2105-7-154-S2.pdf]

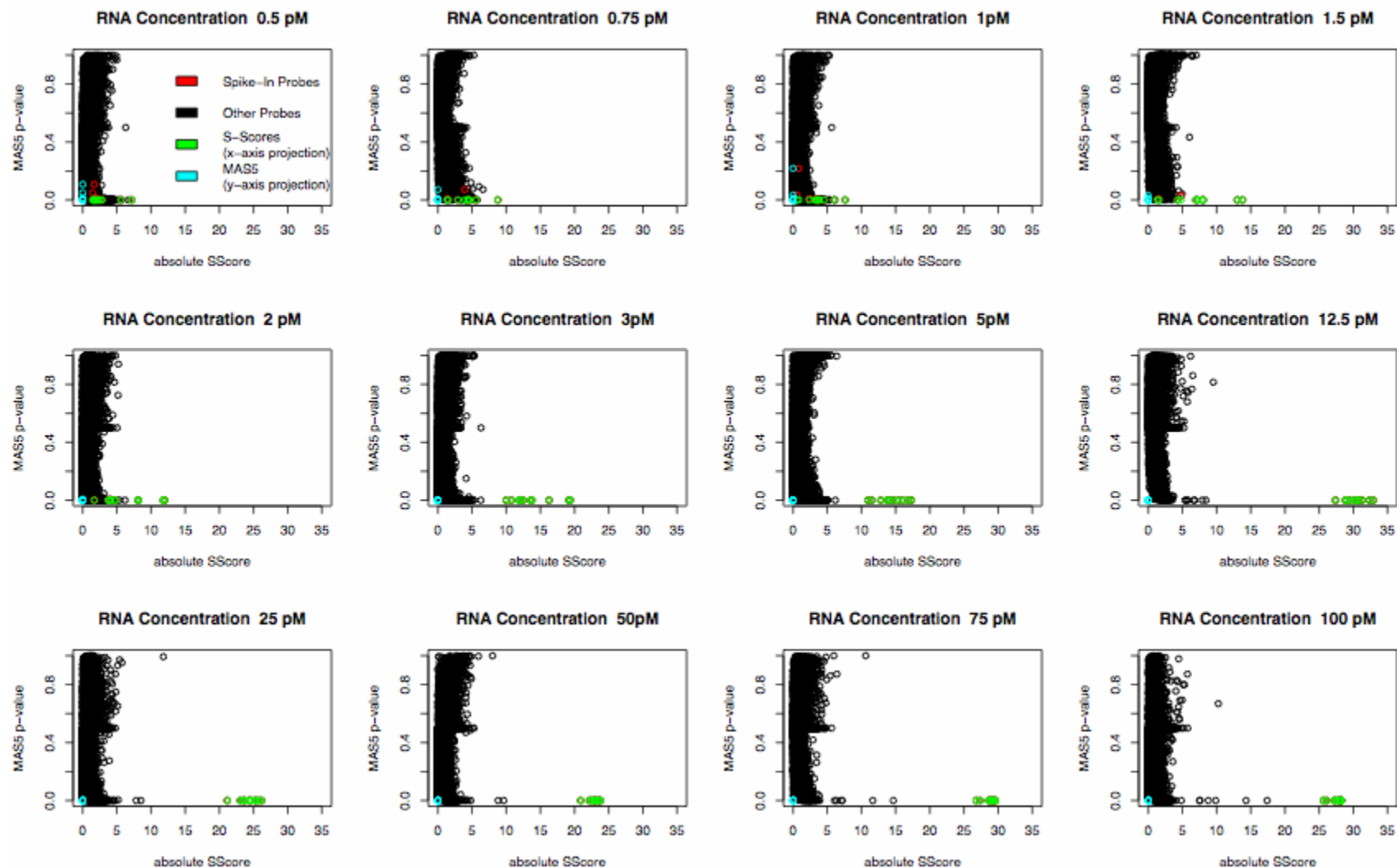

Supplement: Additional File 3 — Comparison of S-Score and MAS5. Plot of absolute value of S-Score vs MAS5 p-values, comparing the specified concentration to the baseline chip. MAS5 p-values were transformed so that significantly up- and down-regulated genes will have p-values approaching 0. X- and Y-axis projections are added to show separation of spike-in probes more clearly. [file 1471-2105-7-154-S3.pdf]
