# Supplementary material for: Empirical validation of the S-Score algorithm in the analysis of gene expression data
Source: BMC Bioinformatics. 2006 Mar 17;7:154. doi: 10.1186/1471-2105-7-154 (PMC1550434; doi:10.1186/1471-2105-7-154)
Supplement: Additional File 5 — Linearity plots for the Latin Square dataset. [file 1471-2105-7-154-S5.pdf]

Concentration vs intensity for chip 92562

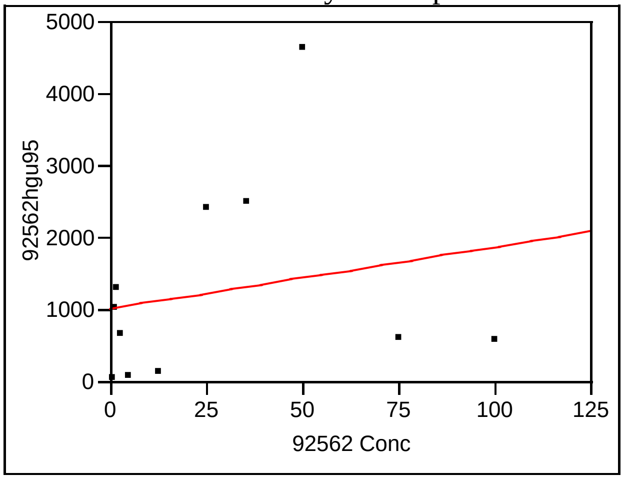

$R^2 = 0.042$

Concentration vs intensity for chip 92558

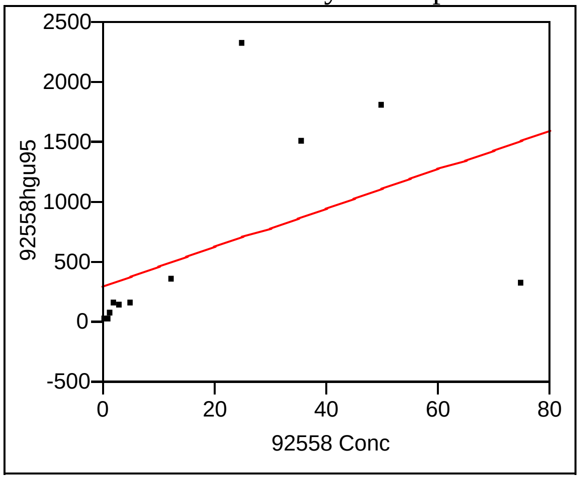

$R^2 = 0.234$

Concentration vs intensity for chip 92563

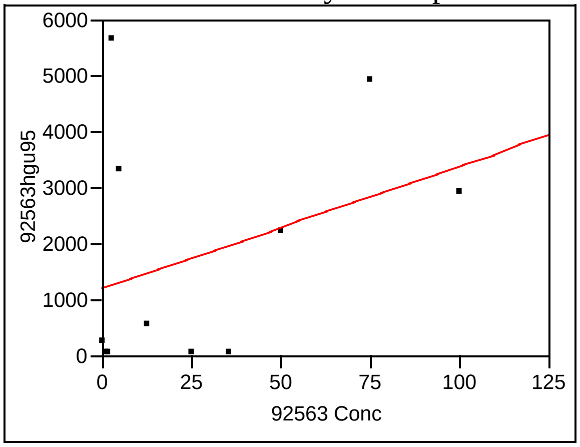

$R^2 = 0.123$

Concentration vs intensity for chip 92559

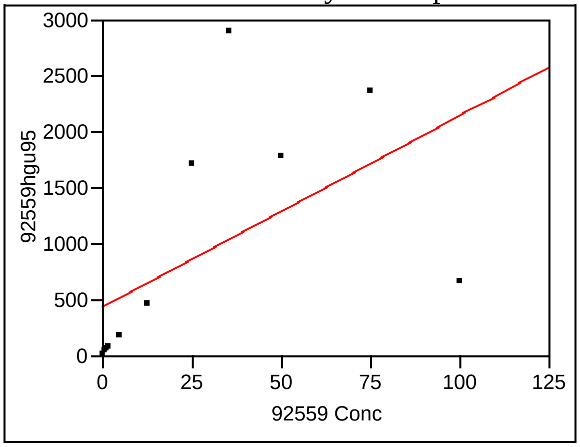

$R^2 = 0.302$

Concentration vs intensity for chip 92564

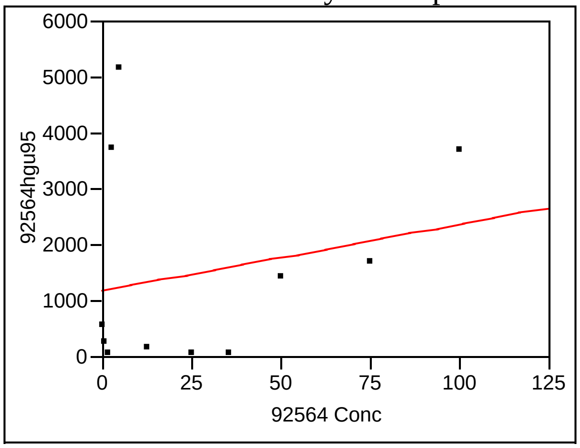

$R^2 = 0.047$

Concentration vs intensity for chip 92560

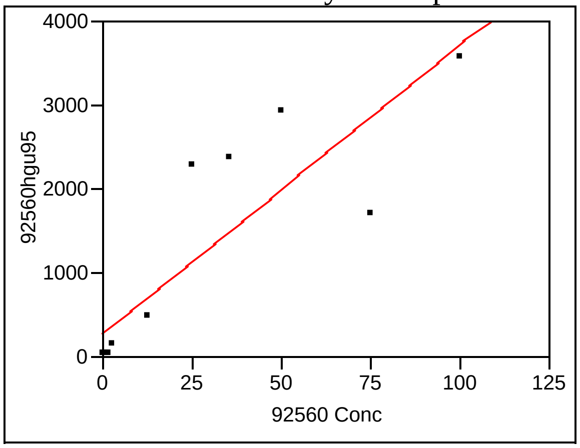

$R^2 = 0.745$

Concentration vs intensity for chip 92554

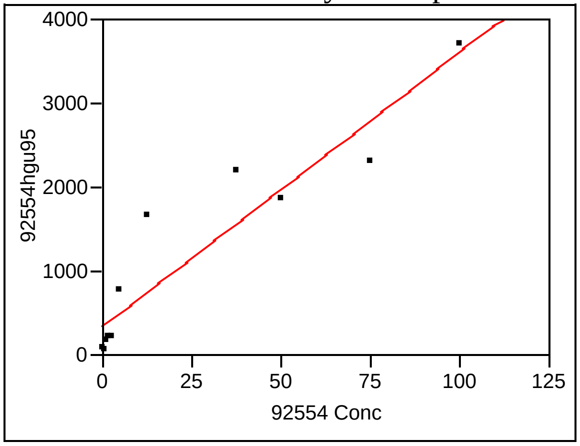

$R^2 = 0.874$

Concentration vs intensity for chip 92556

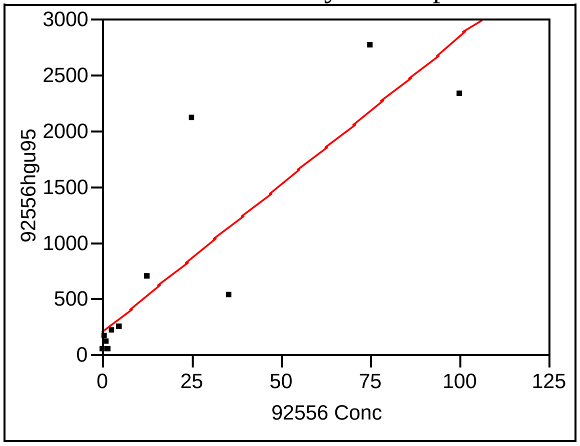

$R^2 = 0.748$

Concentration vs intensity for chip 92555

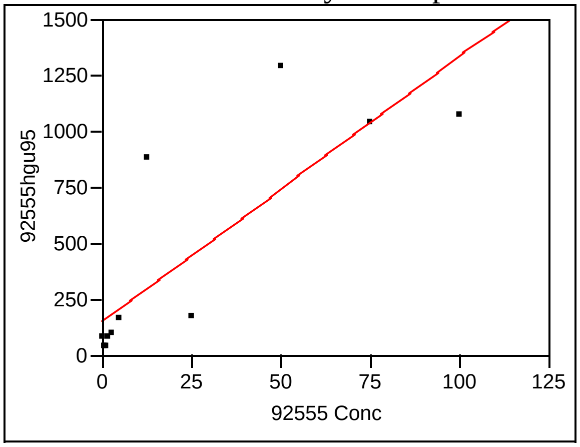

$R^2 = 0.668$

Concentration vs intensity for chip 92557

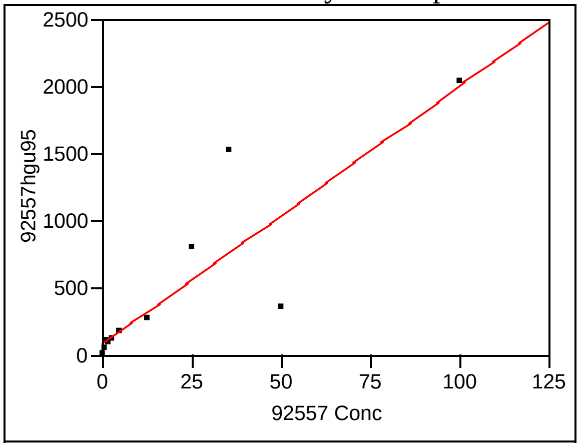

$R^2 = 0.756$
