# Supplementary material for: Empirical validation of the S-Score algorithm in the analysis of gene expression data
Source: BMC Bioinformatics. 2006 Mar 17;7:154. doi: 10.1186/1471-2105-7-154 (PMC1550434; doi:10.1186/1471-2105-7-154)
Supplement: Additional File 6 — Supplementary Tables 1-20. [file 1471-2105-7-154-S6.doc]

**Supplemental Table 1. Concentration data for the Dilution dataset.**

| **GeneChip array** | BioB- **5_at pM** | **BioB-**  **M_at pM** | **BioB-**  **3_at pM** | **BioC-**  **5_at pM** | **BioC-**  **3_at pM** | **BioDn-**  **3_at**  **pM** | **DapX-**  **5_at pM** | **DapX-**  **M_at pM** | **DapX-**  **3_at pM** | **CreX-**  **5_at pM** | **CreX-**  **3_at pM** |
| --- | --- | --- | --- | --- | --- | --- | --- | --- | --- | --- | --- |
| 92466hgu95a11 | 0 | 0 | 0 | 0 | 0 | 0 | 0 | 0 | 0 | 0 | 0 |
| 92491hgu95a11 | 0.5 | 0.5 | 0.5 | 0.5 | 0.5 | 0.5 | 0.5 | 0.5 | 0.5 | 0.5 | 0 |
| 92492hgu95a11 | 0.75 | 0.75 | 0.75 | 0.75 | 0.75 | 0.75 | 0.75 | 0.75 | 0.75 | 0.75 | 0 |
| 92493hgu95a11 | 1 | 1 | 1 | 1 | 1 | 1 | 1 | 1 | 1 | 1 | 0 |
| 92494hgu95a11 | 1.5 | 1.5 | 1.5 | 1.5 | 1.5 | 1.5 | 1.5 | 1.5 | 1.5 | 1.5 | 0 |
| 92495hgu95a11 | 2 | 2 | 2 | 2 | 2 | 2 | 2 | 2 | 2 | 2 | 0 |
| 92496hgu95a11 | 3 | 3 | 3 | 3 | 3 | 3 | 3 | 3 | 3 | 3 | 0 |
| 92453hgu95a11 | 5 | 5 | 5 | 5 | 5 | 5 | 5 | 5 | 5 | 5 | 0 |
| 92497hgu95a11 | 5 | 5 | 5 | 5 | 5 | 5 | 5 | 5 | 5 | 5 | 0 |
| 92454hgu95a11 | 12.5 | 12.5 | 12.5 | 12.5 | 12.5 | 12.5 | 12.5 | 12.5 | 12.5 | 12.5 | 0 |
| 92455hgu95a11 | 12.5 | 12.5 | 12.5 | 12.5 | 12.5 | 12.5 | 12.5 | 12.5 | 12.5 | 12.5 | 0 |
| 92498hgu95a11 | 12.5 | 12.5 | 12.5 | 12.5 | 12.5 | 12.5 | 12.5 | 12.5 | 12.5 | 12.5 | 0 |
| 92456hgu95a11 | 25 | 25 | 25 | 25 | 25 | 25 | 25 | 25 | 25 | 25 | 0 |
| 92457hgu95a11 | 25 | 25 | 25 | 25 | 25 | 25 | 25 | 25 | 25 | 25 | 0 |
| 92499hgu95a11 | 25 | 25 | 25 | 25 | 25 | 25 | 25 | 25 | 25 | 25 | 0 |
| 92458hgu95a11 | 50 | 50 | 50 | 50 | 50 | 50 | 50 | 50 | 50 | 50 | 0 |
| 92459hgu95a11 | 50 | 50 | 50 | 50 | 50 | 50 | 50 | 50 | 50 | 50 | 0 |
| 92500hgu95a11 | 50 | 50 | 50 | 50 | 50 | 50 | 50 | 50 | 50 | 50 | 0 |
| 92460hgu95a11 | 75 | 75 | 75 | 75 | 75 | 75 | 75 | 75 | 75 | 75 | 0 |
| 92461hgu95a11 | 75 | 75 | 75 | 75 | 75 | 75 | 75 | 75 | 75 | 75 | 0 |
| 92501hgu95a11 | 75 | 75 | 75 | 75 | 75 | 75 | 75 | 75 | 75 | 75 | 0 |
| 92462hgu95a11 | 100 | 100 | 100 | 100 | 100 | 100 | 100 | 100 | 100 | 100 | 0 |
| 92463hgu95a11 | 100 | 100 | 100 | 100 | 100 | 100 | 100 | 100 | 100 | 100 | 0 |
| 92464hgu95a11 | 150 | 150 | 150 | 150 | 150 | 150 | 150 | 150 | 150 | 150 | 0 |
| 92465hgu95a11 | 150 | 150 | 150 | 150 | 150 | 150 | 150 | 150 | 150 | 150 | 0 |
| 92503hgu95a11 | 150 | 150 | 150 | 150 | 150 | 150 | 150 | 150 | 150 | 150 | 0 |

**Supplemental Table 2. Concentration data for Latin Square dataset.**

| **GeneChip Array** | **BioB-**  **5_at pM** | **BioB-**  **M_at pM** | **BioB-**  **3_at pM** | **BioC-**  **5_at pM** | **BioC-**  **3_at pM** | **BioDn-**  **3_at**  **pM** | **DapX-**  **5_at pM** | **DapX-**  **M_at pM** | **DapX-**  **3_at pM** | **CreX-**  **5_at pM** | CreX- **3_at pM** |
| --- | --- | --- | --- | --- | --- | --- | --- | --- | --- | --- | --- |
| 92561hgu95a11 | 0.5 | 35.7 | 25 | 75 | 100 | 50 | 1.5 | 1 | 3 | 2 | 5 |
| 92561hgu95a21 | 0.5 | 35.7 | 25 | 75 | 100 | 50 | 1.5 | 1 | 3 | 2 | 5 |
| 92561hgu95a31 | 0.5 | 35.7 | 25 | 75 | 100 | 50 | 1.5 | 1 | 3 | 2 | 5 |
| 92562hgu95a11 | 1 | 50 | 35.7 | 100 | 3 | 75 | 2 | 1.5 | 5 | 25 | 12.5 |
| 92562hgu95a31 | 1 | 50 | 35.7 | 100 | 3 | 75 | 2 | 1.5 | 5 | 25 | 12.5 |
| 92563hgu95a11 | 1.5 | 75 | 50 | 3 | 5 | 100 | 25 | 2 | 12.5 | 35.7 | 0.5 |
| 92563hgu95a21 | 1.5 | 75 | 50 | 3 | 5 | 100 | 25 | 2 | 12.5 | 35.7 | 0.5 |
| 92563hgu95a31 | 1.5 | 75 | 50 | 3 | 5 | 100 | 25 | 2 | 12.5 | 35.7 | 0.5 |
| 92564hgu95a11 | 2 | 100 | 75 | 5 | 12.5 | 3 | 35.7 | 25 | 0.5 | 50 | 1 |
| 92564hgu95a21 | 2 | 100 | 75 | 5 | 12.5 | 3 | 35.7 | 25 | 0.5 | 50 | 1 |
| 92564hgu95a31 | 2 | 100 | 75 | 5 | 12.5 | 3 | 35.7 | 25 | 0.5 | 50 | 1 |
| 92558hgu95a11 | 3 | 1.5 | 1 | 25 | 35.7 | 2 | 12.5 | 5 | 50 | 0.5 | 75 |
| 92558hgu95a21 | 3 | 1.5 | 1 | 25 | 35.7 | 2 | 12.5 | 5 | 50 | 0.5 | 75 |
| 92558hgu95a31 | 3 | 1.5 | 1 | 25 | 35.7 | 2 | 12.5 | 5 | 50 | 0.5 | 75 |
| 92559hgu95a11 | 5 | 2 | 1.5 | 35.7 | 50 | 25 | 0.5 | 12.5 | 75 | 1 | 100 |
| 92559hgu95a21 | 5 | 2 | 1.5 | 35.7 | 50 | 25 | 0.5 | 12.5 | 75 | 1 | 100 |
| 92559hgu95a31 | 5 | 2 | 1.5 | 35.7 | 50 | 25 | 0.5 | 12.5 | 75 | 1 | 100 |
| 92560hgu95a11 | 12.5 | 25 | 2 | 50 | 75 | 35.7 | 1 | 0.5 | 100 | 1.5 | 3 |
| 92560hgu95a21 | 12.5 | 25 | 2 | 50 | 75 | 35.7 | 1 | 0.5 | 100 | 1.5 | 3 |
| 92560hgu95a31 | 12.5 | 25 | 2 | 50 | 75 | 35.7 | 1 | 0.5 | 100 | 1.5 | 3 |
| 92554hgu95a11 | 37.5 | 5 | 3 | 0.5 | 1 | 12.5 | 75 | 50 | 1.5 | 100 | 2 |
| 92554hgu95a21 | 37.5 | 5 | 3 | 0.5 | 1 | 12.5 | 75 | 50 | 1.5 | 100 | 2 |
| 92554hgu95a31 | 37.5 | 5 | 3 | 0.5 | 1 | 12.5 | 75 | 50 | 1.5 | 100 | 2 |
| 92555hgu95a11 | 50 | 12.5 | 5 | 1 | 1.5 | 0.5 | 100 | 75 | 2 | 3 | 25 |
| 92555hgu95a21 | 50 | 12.5 | 5 | 1 | 1.5 | 0.5 | 100 | 75 | 2 | 3 | 25 |
| 92555hgu95a31 | 50 | 12.5 | 5 | 1 | 1.5 | 0.5 | 100 | 75 | 2 | 3 | 25 |
| 92556hgu95a11 | 75 | 0.5 | 12.5 | 1.5 | 2 | 1 | 3 | 100 | 25 | 5 | 35.7 |
| 92556hgu95a21 | 75 | 0.5 | 12.5 | 1.5 | 2 | 1 | 3 | 100 | 25 | 5 | 35.7 |
| 92556hgu95a31 | 75 | 0.5 | 12.5 | 1.5 | 2 | 1 | 3 | 100 | 25 | 5 | 35.7 |
| 92557hgu95a11 | 100 | 1 | 0.5 | 2 | 25 | 1.5 | 5 | 3 | 35.7 | 12.5 | 50 |
| 92557hgu95a21 | 100 | 1 | 0.5 | 2 | 25 | 1.5 | 5 | 3 | 35.7 | 12.5 | 50 |
| 92557hgu95a31 | 100 | 1 | 0.5 | 2 | 25 | 1.5 | 5 | 3 | 35.7 | 12.5 | 50 |

# Supplemental Table 3. Number of spike-in clones detected

# using chip 92554 as baseline

|  | **Clones Detected** | | | |
| --- | --- | --- | --- | --- |
| GeneChip Array | **S-Score** | **RMA** | **dChip** | **MAS5** |
| 92562 | 9 | 7 | 5 | 11 |
| 92563 | 10 | 9 | 9 | 10 |
| 92564 | 10 | 9 | 7 | 10 |
| 92558 | 11 | 10 | 10 | 11 |
| 92559 | 10 | 9 | 8 | 10 |
| 92560 | 10 | 9 | 8 | 10 |
| 92561 | 10 | 8 | 6 | 11 |
| 92555 | 5 | 2 | 2 | 6 |
| 92556 | 7 | 5 | 5 | 7 |
| 92557 | 10 | 8 | 8 | 11 |

Comparison of S-Score vs. RMA, p = 0.01; vs. dChip, p < 0.001; vs. MAS5, p = 0.40

# Supplemental Table 4. Number of spike-in clones detected

# using chip 92555 as baseline

|  | **Clones Detected** | | | |
| --- | --- | --- | --- | --- |
| GeneChip Array | **S-Score** | **RMA** | **dChip** | **MAS5** |
| 92562 | 10 | 5 | 5 | 11 |
| 92563 | 10 | 8 | 8 | 11 |
| 92564 | 9 | 8 | 8 | 10 |
| 92558 | 9 | 8 | 4 | 9 |
| 92559 | 10 | 7 | 7 | 11 |
| 92560 | 10 | 6 | 6 | 11 |
| 92561 | 11 | 9 | 9 | 11 |
| 92554 | 5 | 2 | 2 | 6 |
| 92556 | 4 | 3 | 3 | 4 |
| 92557 | 10 | 8 | 7 | 11 |

Comparison of S-Score vs. RMA, p < 0.001; vs. dChip, p < 0.001; vs. MAS5, p = 0.20

# Supplemental Table 5. Number of spike-in clones detected

# using chip 92556 as baseline

|  | **Clones Detected** | | | |
| --- | --- | --- | --- | --- |
| GeneChip Array | **S-Score** | **RMA** | **dChip** | **MAS5** |
| 92562 | 10 | 9 | 7 | 11 |
| 92563 | 11 | 9 | 6 | 11 |
| 92564 | 11 | 9 | 6 | 11 |
| 92558 | 8 | 6 | 5 | 8 |
| 92559 | 9 | 8 | 8 | 11 |
| 92560 | 11 | 11 | 9 | 11 |
| 92561 | 10 | 9 | 7 | 11 |
| 92554 | 7 | 5 | 5 | 7 |
| 92555 | 4 | 3 | 3 | 4 |
| 92557 | 9 | 3 | 3 | 9 |

Comparison of S-Score vs. RMA, p = 0.004; vs. dChip, p < 0.001; vs. MAS5, p = 0.53

# Supplemental Table 6. Number of spike-in clones detected

# using chip 92557 as baseline

|  | **Clones Detected** | | | |
| --- | --- | --- | --- | --- |
| GeneChip Array | **S-Score** | **RMA** | **dChip** | **MAS5** |
| 92562 | 10 | 6 | 6 | 11 |
| 92563 | 10 | 5 | 5 | 10 |
| 92564 | 10 | 5 | 5 | 11 |
| 92558 | 5 | 3 | 3 | 5 |
| 92559 | 6 | 5 | 5 | 6 |
| 92560 | 8 | 5 | 5 | 8 |
| 92561 | 10 | 5 | 5 | 11 |
| 92554 | 10 | 8 | 8 | 11 |
| 92555 | 11 | 8 | 7 | 11 |
| 92556 | 9 | 3 | 3 | 9 |

Comparison of S-Score vs. RMA, p < 0.001; vs. dChip, p < 0.001; vs. MAS5, p = 0.55

# Supplemental Table 7. Number of spike-in clones detected

# using chip 92558 as baseline

|  | **Clones Detected** | | | |
| --- | --- | --- | --- | --- |
| GeneChip Array | **S-Score** | **RMA** | **dChip** | **MAS5** |
| 92562 | 10 | 9 | 7 | 11 |
| 92563 | 8 | 5 | 5 | 9 |
| 92564 | 10 | 8 | 6 | 10 |
| 92559 | 5 | 5 | 3 | 5 |
| 92560 | 8 | 8 | 6 | 8 |
| 92561 | 10 | 10 | 8 | 11 |
| 92554 | 11 | 10 | 10 | 11 |
| 92555 | 9 | 8 | 7 | 9 |
| 92556 | 8 | 6 | 6 | 8 |
| 92557 | 5 | 3 | 3 | 5 |

Comparison of S-Score vs. RMA, p = 0.08; vs. dChip, p < 0.001; vs. MAS5, p = 0.72

# Supplemental Table 8. Number of spike-in clones detected

# using chip 92559 as baseline

|  | **Clones Detected** | | | |
| --- | --- | --- | --- | --- |
| GeneChip Array | **S-Score** | **RMA** | **dChip** | **MAS5** |
| 92562 | 10 | 8 | 6 | 11 |
| 92563 | 6 | 5 | 5 | 7 |
| 92564 | 8 | 8 | 7 | 11 |
| 92558 | 5 | 5 | 3 | 5 |
| 92560 | 6 | 5 | 3 | 7 |
| 92561 | 11 | 9 | 9 | 11 |
| 92554 | 10 | 9 | 8 | 11 |
| 92555 | 10 | 7 | 7 | 11 |
| 92556 | 9 | 8 | 8 | 11 |
| 92557 | 6 | 5 | 5 | 6 |

Comparison of S-Score vs. RMA, p = 0.10; vs. dChip, p = 0.005; vs. MAS5, p = 0.10

# Supplemental Table 9. Number of spike-in clones detected

# using chip 92560 as baseline

|  | **Clones Detected** | | | |
| --- | --- | --- | --- | --- |
| GeneChip Array | **S-Score** | **RMA** | **dChip** | **MAS5** |
| 92562 | 10 | 8 | 8 | 11 |
| 92563 | 3 | 3 | 3 | 5 |
| 92564 | 5 | 5 | 5 | 6 |
| 92558 | 8 | 8 | 6 | 8 |
| 92559 | 6 | 5 | 3 | 7 |
| 92561 | 9 | 7 | 7 | 9 |
| 92554 | 10 | 9 | 8 | 10 |
| 92555 | 10 | 6 | 6 | 11 |
| 92556 | 11 | 11 | 9 | 11 |
| 92557 | 8 | 5 | 5 | 8 |

Comparison of S-Score vs. RMA, p = 0.06; vs. dChip, p = 0.004; vs. MAS5, p = 0.39

# Supplemental Table 10. Number of spike-in clones detected

# using chip 92562 as baseline

|  | **Clones Detected** | | | |
| --- | --- | --- | --- | --- |
| GeneChip Array | **S-Score** | **RMA** | **dChip** | **MAS5** |
| 92563 | 8 | 7 | 7 | 8 |
| 92564 | 10 | 8 | 6 | 10 |
| 92558 | 10 | 9 | 7 | 11 |
| 92559 | 10 | 8 | 6 | 11 |
| 92560 | 10 | 8 | 8 | 11 |
| 92561 | 4 | 4 | 4 | 4 |
| 92554 | 10 | 7 | 5 | 11 |
| 92555 | 10 | 5 | 5 | 11 |
| 92556 | 10 | 9 | 7 | 11 |
| 92557 | 10 | 6 | 6 | 11 |

Comparison of S-Score vs. RMA, p = 0.001; vs. dChip, p < 0.001; vs. MAS5, p = 0.17

# Supplemental Table 11. Number of spike-in clones detected

# using chip 92563 as baseline

|  | **Clones Detected** | | | |
| --- | --- | --- | --- | --- |
| GeneChip Array | **S-Score** | **RMA** | **dChip** | **MAS5** |
| 92562 | 8 | 7 | 7 | 8 |
| 92564 | 3 | 2 | 1 | 3 |
| 92558 | 8 | 5 | 5 | 9 |
| 92559 | 6 | 5 | 5 | 7 |
| 92560 | 3 | 3 | 3 | 5 |
| 92561 | 8 | 4 | 4 | 7 |
| 92554 | 10 | 9 | 9 | 11 |
| 92555 | 10 | 8 | 8 | 11 |
| 92556 | 11 | 9 | 6 | 11 |
| 92557 | 10 | 5 | 5 | 10 |

Comparison of S-Score vs. RMA, p = 0.004; vs. dChip, p < 0.001; vs. MAS5, p = 0.49

# Supplemental Table 12. Number of spike-in clones detected

# using chip 92564 as baseline

|  | **Clones Detected** | | | |
| --- | --- | --- | --- | --- |
| GeneChip Array | **S-Score** | **RMA** | **dChip** | **MAS5** |
| 92562 | 10 | 8 | 6 | 10 |
| 92563 | 3 | 2 | 2 | 3 |
| 92558 | 10 | 8 | 6 | 10 |
| 92559 | 8 | 8 | 7 | 11 |
| 92560 | 5 | 5 | 5 | 6 |
| 92561 | 8 | 2 | 2 | 8 |
| 92554 | 10 | 9 | 7 | 10 |
| 92555 | 9 | 8 | 8 | 10 |
| 92556 | 10 | 9 | 6 | 11 |
| 92557 | 10 | 5 | 5 | 11 |

Comparison of S-Score vs. RMA, p = 0.005; vs. dChip, p < 0.001; vs. MAS5, p = 0.26

# Supplemental Table 13. Number of spike-in clones detected

# using chip 92554 as baseline with chips 92562-92564 excluded

|  | **Clones Detected** | | | |
| --- | --- | --- | --- | --- |
| GeneChip Array | **S-Score** | **RMA** | **dChip** | **MAS5** |
| 92558 | 11 | 11 | 10 | 11 |
| 92559 | 10 | 10 | 8 | 10 |
| 92560 | 10 | 10 | 7 | 10 |
| 92561 | 10 | 10 | 8 | 11 |
| 92555 | 5 | 6 | 2 | 6 |
| 92556 | 7 | 7 | 5 | 7 |
| 92557 | 11 | 11 | 8 | 11 |

Comparison of S-Score vs. RMA, p > 0.99; vs. dChip, p = 0.003; vs. MAS5, p = 0.80

# Supplemental Table 14. Number of spike-in clones detected

# using chip 92555 as baseline with chips 92562-92564 excluded

|  | **Clones Detected** | | | |
| --- | --- | --- | --- | --- |
| GeneChip Array | **S-Score** | **RMA** | **dChip** | **MAS5** |
| 92558 | 9 | 9 | 7 | 9 |
| 92559 | 10 | 10 | 7 | 11 |
| 92560 | 10 | 10 | 6 | 11 |
| 92561 | 11 | 11 | 9 | 11 |
| 92554 | 5 | 6 | 2 | 6 |
| 92556 | 4 | 4 | 3 | 4 |
| 92557 | 10 | 10 | 6 | 11 |

Comparison of S-Score vs. RMA, p > 0.99; vs. dChip, p < 0.001; vs. MAS5, p = 0.47

# Supplemental Table 15. Number of spike-in clones detected

# using chip 92556 as baseline with chips 92562-92564 excluded

|  | **Clones Detected** | | | |
| --- | --- | --- | --- | --- |
| GeneChip Array | **S-Score** | **RMA** | **dChip** | **MAS5** |
| 92558 | 8 | 8 | 1 | 8 |
| 92559 | 9 | 11 | 8 | 11 |
| 92560 | 11 | 11 | 9 | 11 |
| 92561 | 10 | 10 | 6 | 11 |
| 92554 | 7 | 7 | 5 | 7 |
| 92555 | 4 | 4 | 3 | 4 |
| 92557 | 9 | 11 | 3 | 9 |

Comparison of S-Score vs. RMA, p = 0.51; vs. dChip, p < 0.001; vs. MAS5, p = 0.67

# Supplemental Table 16. Number of spike-in clones detected

# using chip 92557 as baseline with chips 92562-92564 excluded

|  | **Clones Detected** | | | |
| --- | --- | --- | --- | --- |
| GeneChip Array | **S-Score** | **RMA** | **dChip** | **MAS5** |
| 92558 | 5 | 4 | 3 | 5 |
| 92559 | 6 | 6 | 5 | 6 |
| 92560 | 8 | 8 | 5 | 8 |
| 92561 | 10 | 10 | 5 | 11 |
| 92554 | 11 | 11 | 8 | 11 |
| 92555 | 10 | 10 | 6 | 11 |
| 92556 | 9 | 11 | 3 | 9 |

Comparison of S-Score vs. RMA, p > 0.99; vs. dChip, p < 0.001; vs. MAS5, p = 0.83

# Supplemental Table 17. Number of spike-in clones detected

# using chip 92558 as baseline with chips 92562-92564 excluded

|  | **Clones Detected** | | | |
| --- | --- | --- | --- | --- |
| GeneChip Array | **S-Score** | **RMA** | **dChip** | **MAS5** |
| 92559 | 5 | 4 | 3 | 5 |
| 92560 | 8 | 8 | 6 | 8 |
| 92561 | 10 | 11 | 8 | 11 |
| 92554 | 11 | 11 | 10 | 11 |
| 92555 | 9 | 9 | 7 | 9 |
| 92556 | 8 | 8 | 6 | 8 |
| 92557 | 5 | 4 | 3 | 5 |

Comparison of S-Score vs. RMA, p > 0.99; vs. dChip, p = 0.03; vs. MAS5, p > 0.99

# Supplemental Table 18. Number of spike-in clones detected

# using chip 92559 as baseline with chips 92562-92564 excluded

|  | **Clones Detected** | | | |
| --- | --- | --- | --- | --- |
| GeneChip Array | **S-Score** | **RMA** | **dChip** | **MAS5** |
| 92558 | 5 | 4 | 3 | 5 |
| 92560 | 6 | 6 | 3 | 7 |
| 92561 | 11 | 11 | 9 | 11 |
| 92554 | 10 | 10 | 8 | 10 |
| 92555 | 10 | 10 | 7 | 11 |
| 92556 | 9 | 11 | 8 | 11 |
| 92557 | 6 | 6 | 5 | 6 |

Comparison of S-Score vs. RMA, p = 0.84; vs. dChip, p = 0.02; vs. MAS5, p = 0.52

# Supplemental Table 19. Number of spike-in clones detected

# using chip 92560 as baseline with chips 92562-92564 excluded

|  | **Clones Detected** | | | |
| --- | --- | --- | --- | --- |
| GeneChip Array | **S-Score** | **RMA** | **dChip** | **MAS5** |
| 92558 | 8 | 8 | 6 | 8 |
| 92559 | 6 | 6 | 3 | 7 |
| 92561 | 9 | 9 | 7 | 9 |
| 92554 | 10 | 10 | 6 | 10 |
| 92555 | 10 | 10 | 6 | 11 |
| 92556 | 11 | 11 | 9 | 11 |
| 92557 | 8 | 8 | 5 | 8 |

Comparison of S-Score vs. RMA, p = 0.83; vs. dChip, p < 0.001; vs. MAS5, p = 0.83

# Supplemental Table 20. Number of spike-in clones detected

# using chip 92561 as baseline with chips 92562-92564 excluded

|  | **Clones Detected** | | | |
| --- | --- | --- | --- | --- |
| GeneChip Array | **S-Score** | **RMA** | **dChip** | **MAS5** |
| 92558 | 10 | 11 | 8 | 11 |
| 92559 | 11 | 11 | 8 | 11 |
| 92560 | 9 | 9 | 7 | 9 |
| 92554 | 10 | 10 | 8 | 11 |
| 92555 | 11 | 11 | 9 | 11 |
| 92556 | 10 | 10 | 5 | 11 |
| 92557 | 10 | 10 | 5 | 11 |

Comparison of S-Score vs. RMA, p > 0.99; vs. dChip, p < 0.001; vs. MAS5, p = 0.27
